# Supplementary material for: Pulmonary inflammation promoted by type-2 dendritic cells is a feature of human and murine schistosomiasis
Source: Nat Commun. 2023 Apr 3;14:1863. doi: 10.1038/s41467-023-37502-z (PMC10070318; doi:10.1038/s41467-023-37502-z)
Supplement: Supplementary file 1 — Supplementary Information [file 41467_2023_37502_MOESM1_ESM.pdf]

	Participants (n=3)
**Study design**	Clinical trial
**Cercarial dose**	20
**Sputum sampling time post infection**	11 - 14 days
**Study location**	Leiden, Netherlands
**Ages**	18 - 35 years
**Sex**	2 Male, 1 Female

Supplementary Figure 1. Participant characteristics - pre-patent controlled human schistosome infection study.

	Control (n=15)	Case (n=12)
**Study design**	Observational, case control	
Infectious cercarial dose and time post first infection unknown		
**Study location**	Kigungu, Entebbe, Uganda	
Participants were recruited within a 2km radius of the Kigungu landing site controls from colleges, cases from the community		
**Age – median (min, max)**	19.5 (18, 23)	19 (18, 25)
**Sex - male, number (% of total)**	8 (53%)	7 (58%)
**Student - yes, number (% of total)**	12 (80%)	1 (8%)
**Ever done work activities involving regular lake contact?**		
Yes	6 (38%)	11 (92%)
currently/last month	2 (13%)	9 (75%)
two-six months ago	3 (19%)	1 (8%)
seven-twelve months ago	1 (6%)	0 (0%)
more than a year ago	0 (0%)	1 (8%)
unknown	0 (0%)	0 (0%)
No	9 (56%)	1 (8%)
Unknown	0 (0%)	0 (0%)
**Kato Katz - positive, number (% of total)**		
S. mansoni	0% (0±0)	12 (100%)
Light (0-99 eggs/g)	N/A	4 (33%)
Moderate (100-199 eggs/g)	N/A	4 (33%)
Heavy (199-200 eggs/g)	N/A	4 (33%)
No other worm eggs (Ascaris, Trichuris, Strongyloides, Trichostrongylus, S. haematobium, or Hookworm) found		
**Last praziquantel dose - number (% of total)**		
in the last month	1 (6.3%)	0 (0%)
two to six months ago	0 (0%)	0 (0%)
seven to twelve months ago	0 (0%)	1 (8%)
more than a year ago	6 (37.5%)	2 (17%)
not at all	2 (12.5%)	8 (67%)
do not remember/not recorded	6 (37.5%)	1 (8%)

Supplementary Figure 2. Participant characteristics - endemic patent infection study

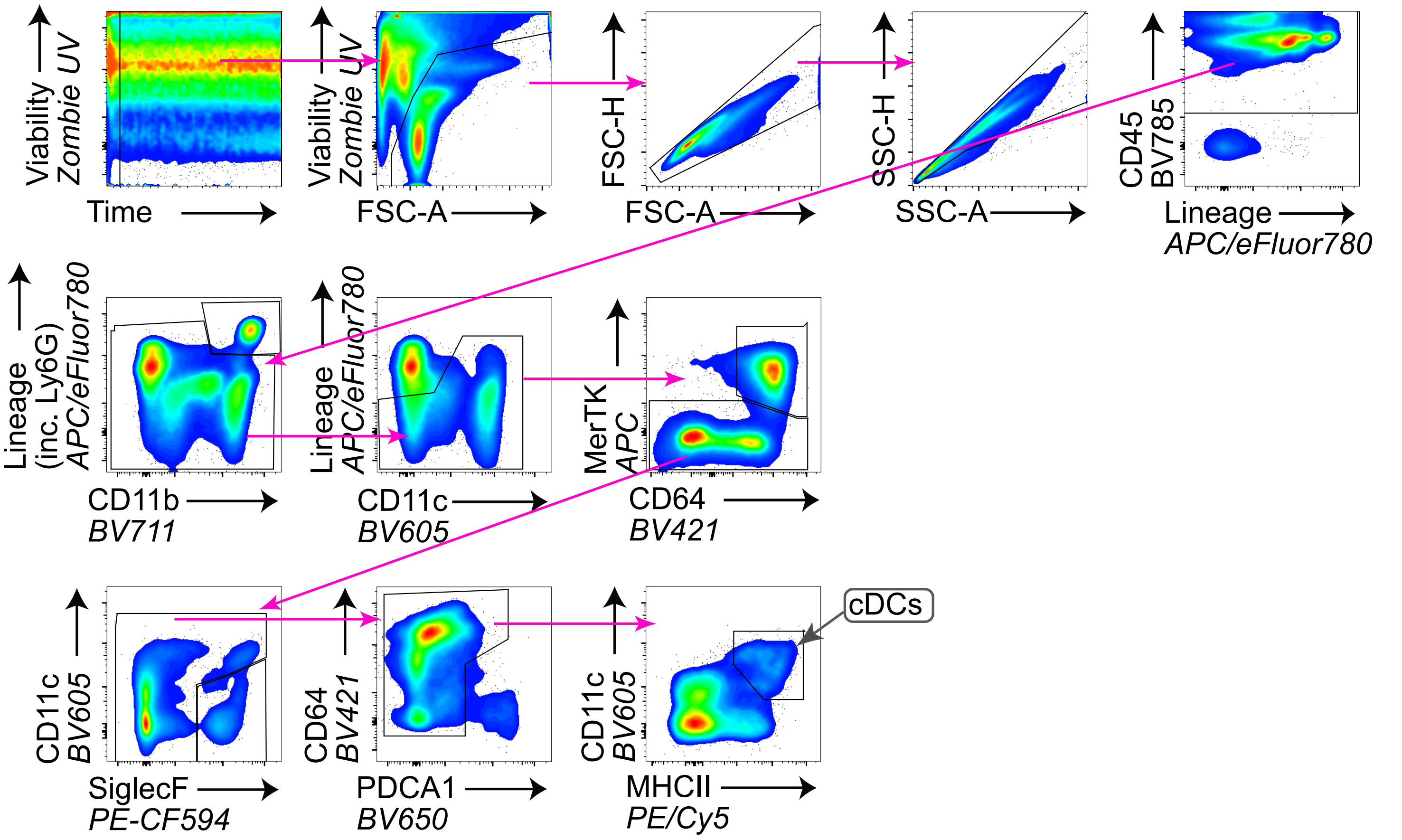

Supplementary Figure 3. Gating scheme murine cDCs. Lung cell isolates were assessed via flow cytometry to define cDCs via the gating strategy shown. Lineage gate includes Ter119 APC-e780, CD3 APC-e780, NK1.1 APC-e780, Ly6G APC-Cy7 and CD19-APC-e780. Live, singlet, CD45^{+} cells were assessed, with numerous cell populations, including MerTK^{+} CD64^{+} macrophages gated out prior to identification of CD11c^{+} MHCII^{+} cDCs.

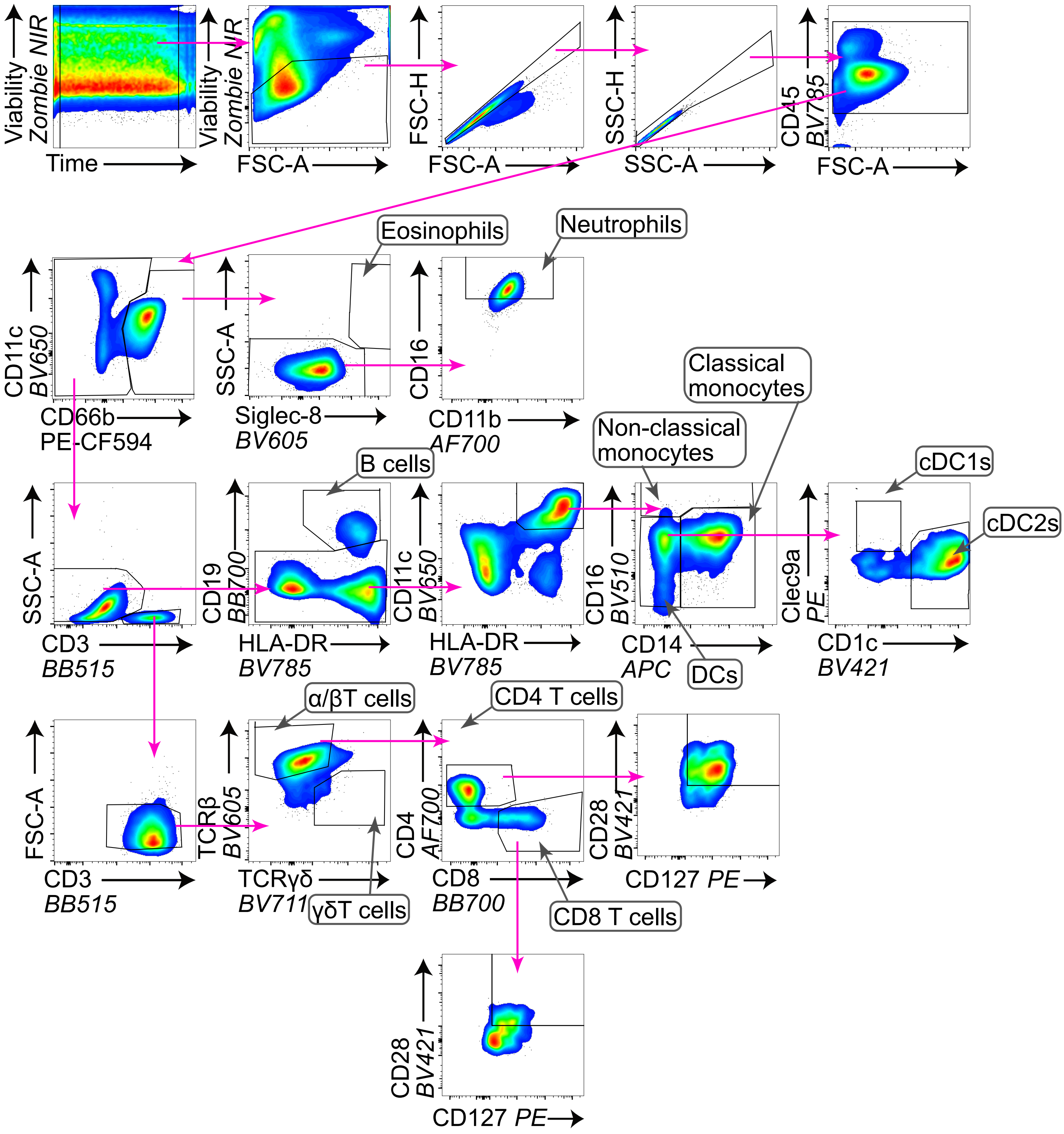

Supplementary Figure 4. Gating scheme human sputum immune populations. Plots are taken from one representative individual in the patent endemic *S. mansoni* infection study, and are representative of all sputum gating. Sentinel gating was used, with the following markers sharing a fluorescent channel: CD8 & CD19, CD4 & CD11b, CD28 & CD1c, TCRβ & Siglec 8, TCRγδ & CD14 or HLA-DR (study dependent) as well as CD127 & Clec9a.

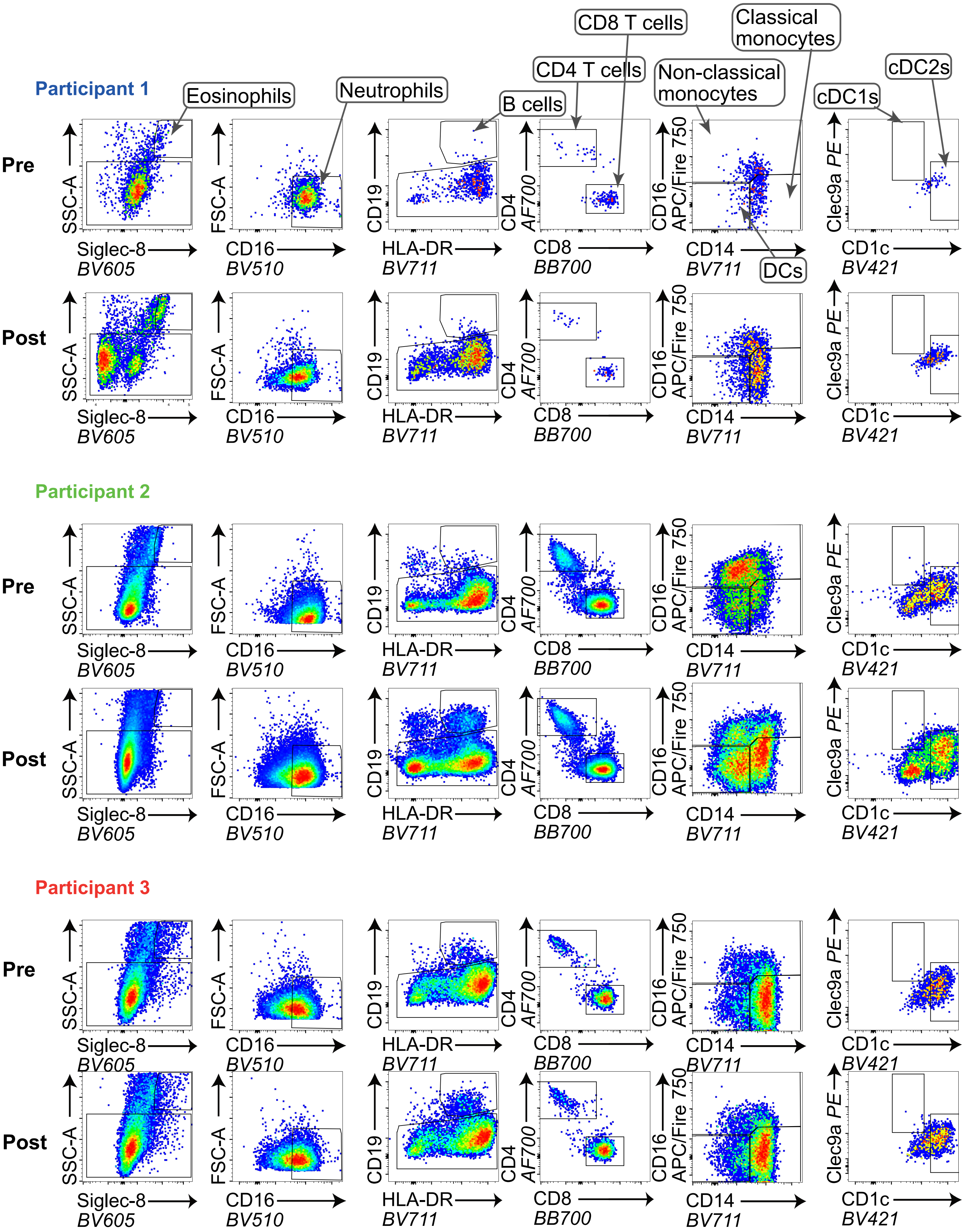

Supplementary Figure 5. Sputum flow cytometry plots from all participants pre and 11-14 days post percutaneous infection with 20 cercariae, during pre-patent infection. Plots are taken from all individuals measured in the pre-patent endemic *S. mansoni* infection sputum study. Gating scheme detailed in Supp. Fig. 4. Sentinel gating was used, with some fluorescent channels including two antibodies, as detailed in methods. Colours denote individual participants.

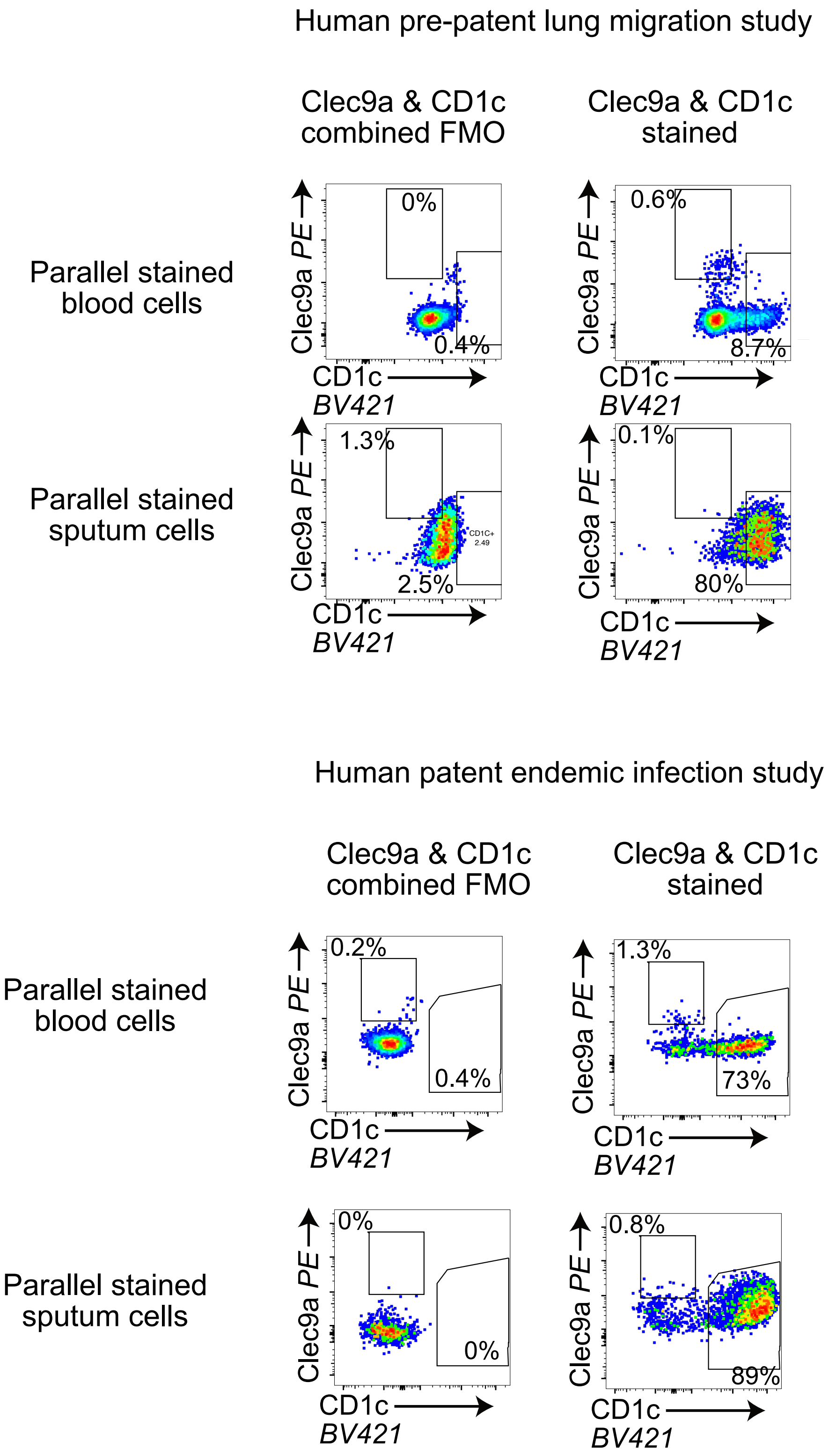

Supplementary Figure 6. CD1c and Clec9a staining in human sputum and blood cDCs. In lung migratory and patent human infection studies sputum and healthy control blood samples were run in parallel, with FMOs used to define gate positions. Representative flow cytometry plots are pre-gated on cDCs (HLA-DR$^+$ CD11c$^+$ CD14$^-$ CD16$^-$), as shown in Supp. Fig. 3. Frequencies on gate are % of cDCs.

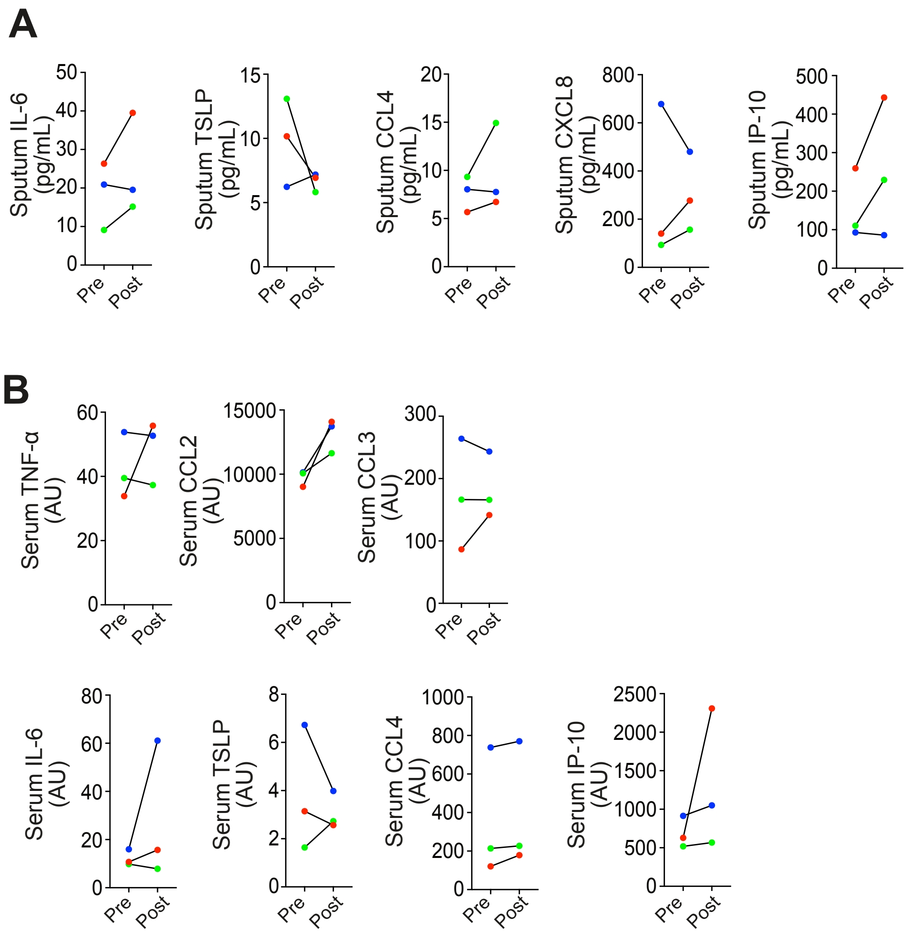

Supplementary Figure 7. Sputum and serum inflammatory mediators - human pre-patent infection. Pulmonary and systemic responses were studied in non-endemic participants in Leiden, Netherlands infected percutaneously with 20 *S. mansoni* cercariae, with induced sputum samples taken pre, and 11-14 days post infection. A) Inflammatory mediators in sputum supernatants were assessed by Luminex. B) Inflammatory mediators in serum were assessed by Olink, expressed in Arbitrary Units (AU), at timepoint 0 and at 2 weeks post infection. Colours denote individual participants. Data are from one study (n=6), two-sided paired t tests were used to compare differences between groups. Serum levels of the following cytokines were not measured: IL-1β, IL-1RA, CCL17, YKL-40, CCL22 and CXCL8. Source data are provided as a Source Data file.

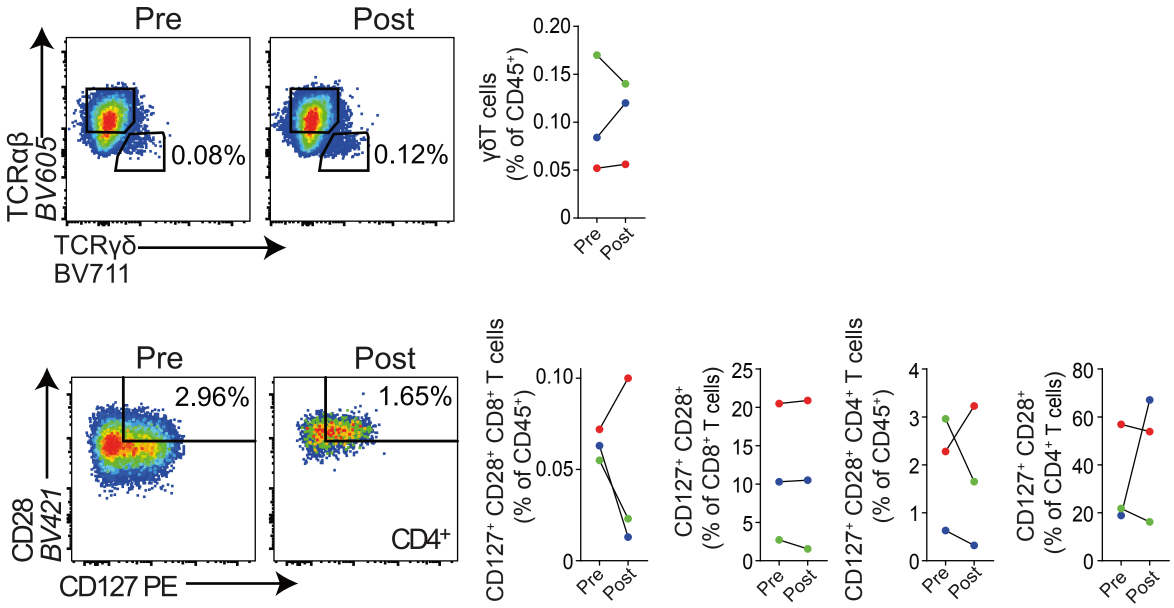

Supplementary Figure 8. Sputum γδ T cell frequency and αβ T cell activation - human pre-patent infection. Sputum cells were isolated, and assessed by flow cytometry as in Supp. Fig. 3. Representative flow cytometry plots show changes in γδ T cells, and activation of αβ T cells. Gate frequencies show % of $CD45^+$ cells. Colours denote individual participants. Data are from one study (n=6), two-sided paired T tests were used to compare differences between groups. Source data are provided as a Source Data file.

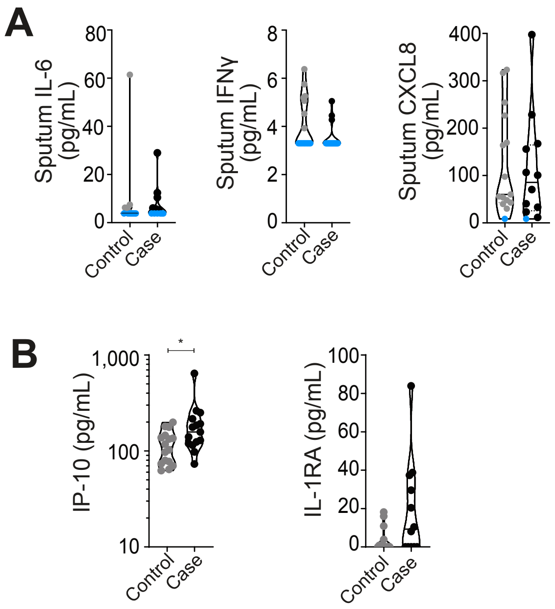

Supplementary Figure 9. Sputum and serum inflammatory mediators - human endemic patent infection. Pulmonary and systemic responses in uninfected control, and *S. mansoni* infected cases from Entebbe, Uganda were compared. Inflammatory mediators in A) sputum supernatants and B) serum were assessed by Luminex. Samples below the detection limit were assigned the value of the lowest standard, and are shown in blue. Data are from one study (n=27 individuals), two-sided Mann-Whitney tests were used to compare differences between groups. The following cytokines were below the limit of detection in serum so were not included: IL-1β, IL-6, IFNγ, CXCL8. Source data are provided as a Source Data file.

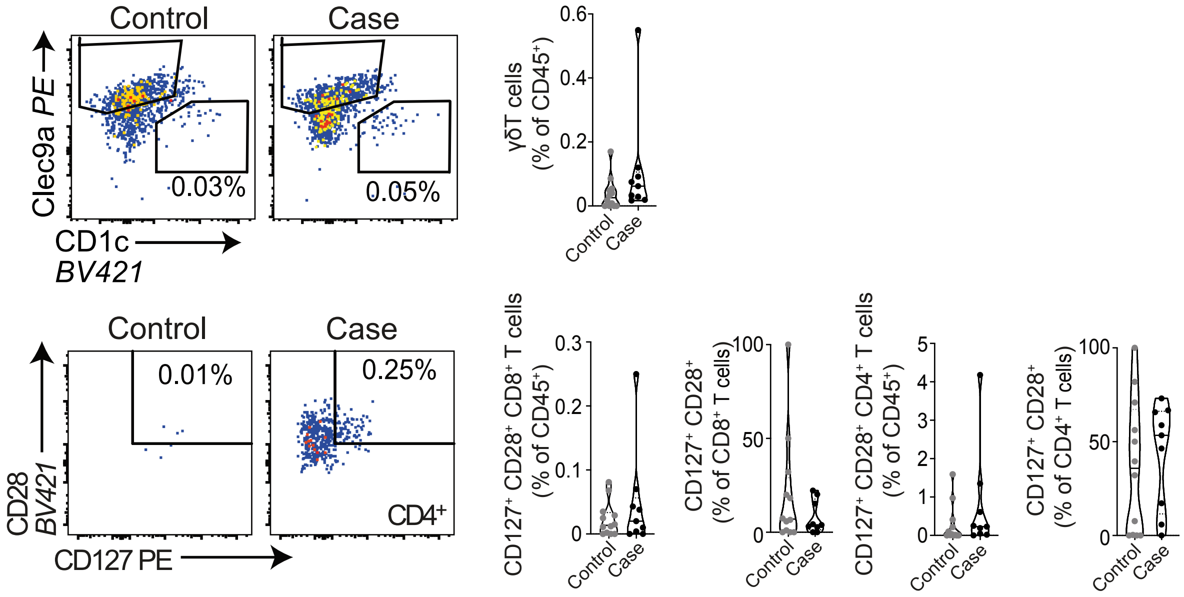

Supplementary Figure 10. Sputum γδ T cell frequency and αβ T cell activation and - human endemic patent infection. Sputum cells were isolated, and assessed by flow cytometry as in Supp. Fig. 4. Representative flow cytometry plots show changes in γδ T cells, and activation of αβ T cells. Gate frequencies show % of $CD45^+$ cells. Data are from one study (n=27 individuals), two-sided Mann-Whitney tests were used to compare differences between groups. Source data are provided as a Source Data file.

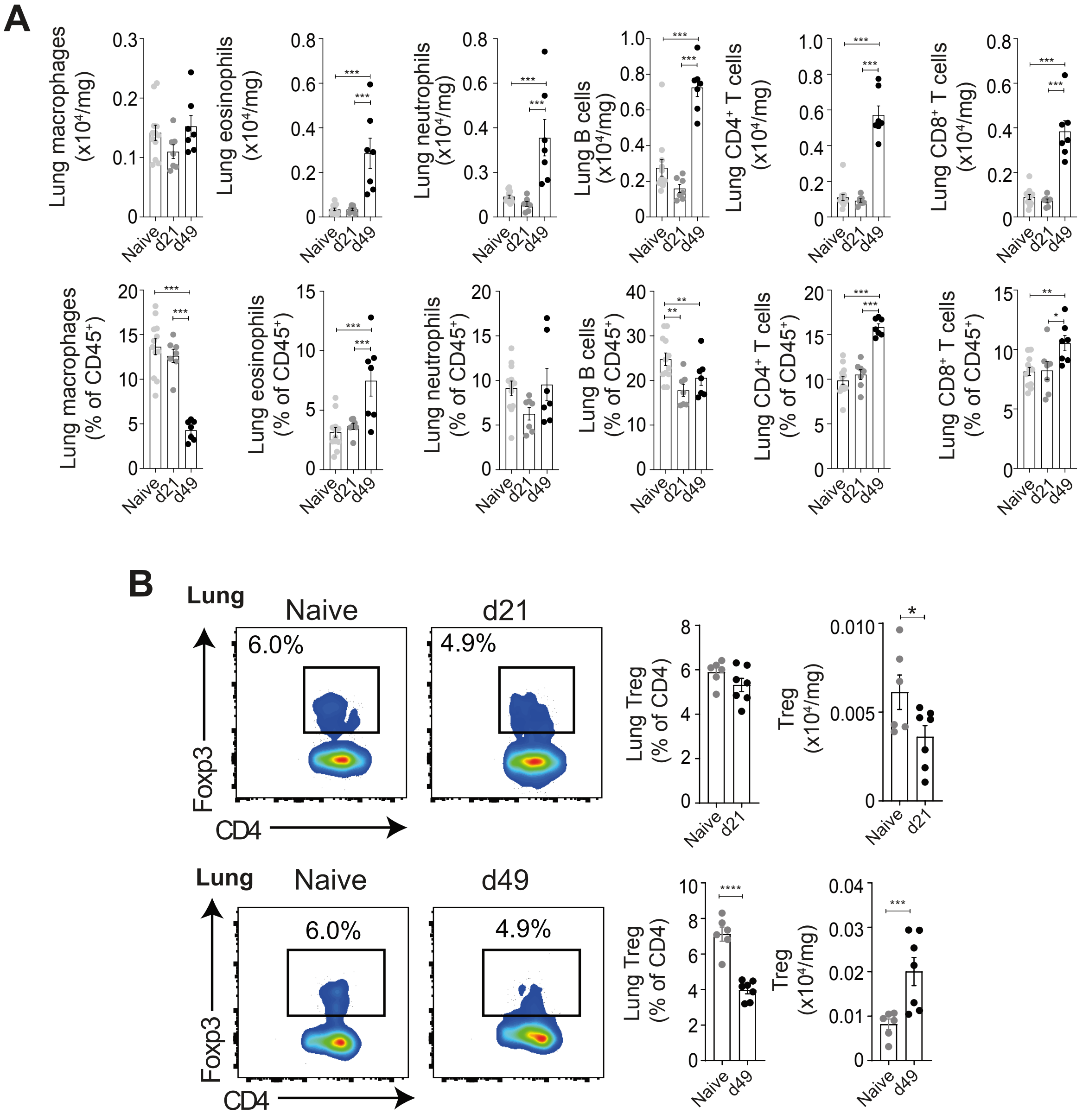

Supplementary Figure 11 - Lung immune cell changes in lung migratory and patent murine schistosome infection. C57BL/6 mice were percutaneously infected with 180 cercariae, and pulmonary samples taken at d21 and d49. A) Lung cells were isolated, and assessed by flow cytometry for eosinophils, neutrophils, B cells, $CD4^+$ and $CD8^+$ T cells. B) Representative flow cytometry plots depict gating of lung Tregs, post PMA/ionomycin stimulation. Data are from 3 experiments, (n= 19 biologically independent animals). Data were fit to a linear mixed effect model, with experimental day as a random effect variable, and groups compared with a two-sided Tukey's multiple comparison test. *=P <0.05, **=p<0.01, ***=p<0.001, ****=p<0.0001. Data are presented as mean values +/- SEM. Source data are provided as a Source Data file.

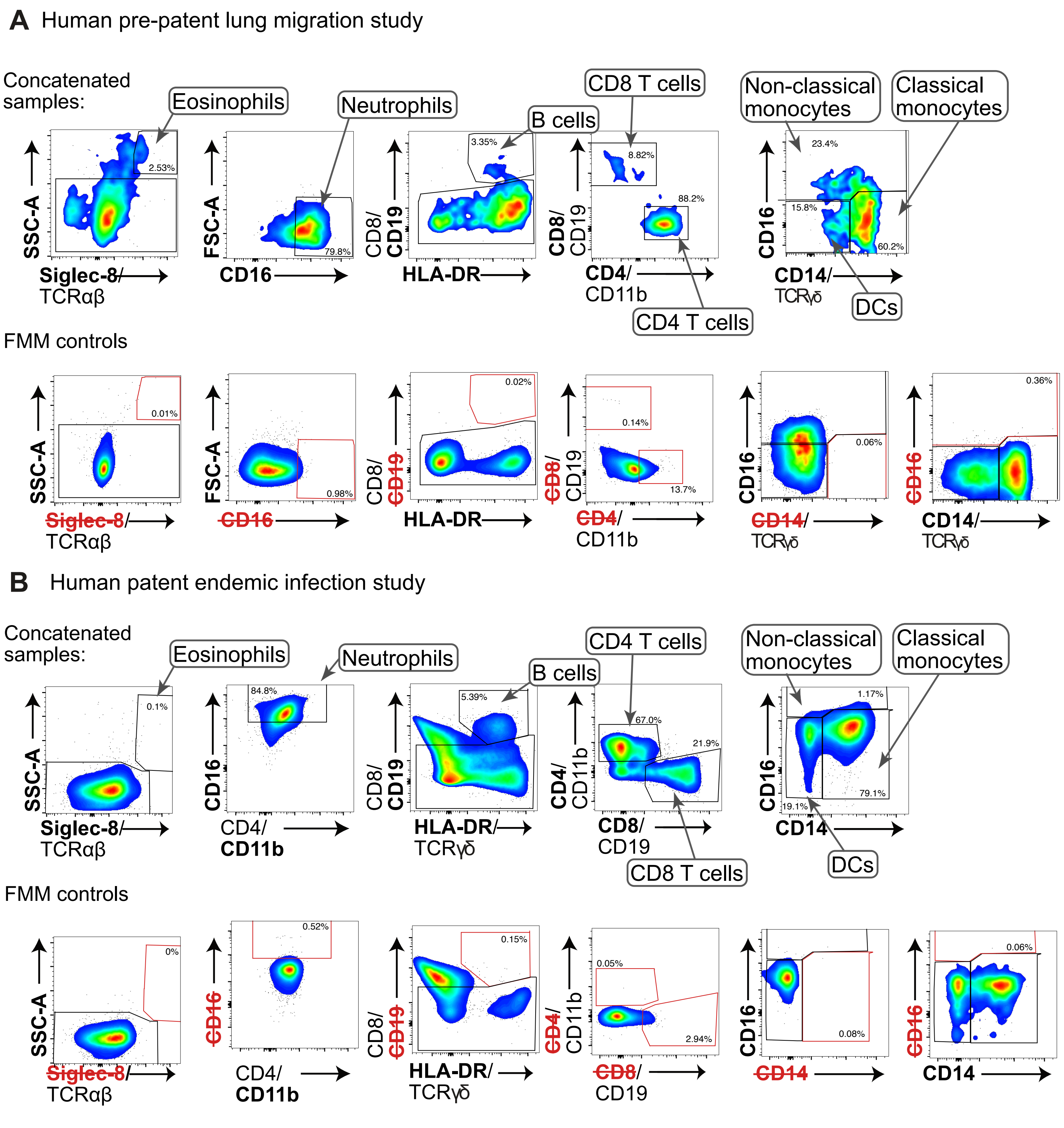

Supplementary Figure 12 - Concatenated plots and controls from pre-patent and endemic patent sputum flow cytometry. A) Pre-patent infection, B) Endemic patent infection. Plots shown are either concatenated plots from all samples, or from relevant fluorescence minus many (FMM) controls. Gating scheme seen in Supplementary Figure 4, Clec9a and CD1c staining in Supplementary Figure 6.

Sentinel gating was used, with the following markers sharing a fluorescent channel: CD8 & CD19, CD4 & CD11b, CD28 & CD1c, TCRβ & Siglec 8, TCRγδ & CD14 or HLA-DR (study dependent) as well as CD127 & Clec9a. The antigen used to identify the cell type indicated is in **bold**.

Due to limitations in sputum cell number FMM controls were used, with 4-5 antibodies (out of 22 total) missing in each control sample. When an antibody was not added this is indicated by in **red** with a strikethrough.

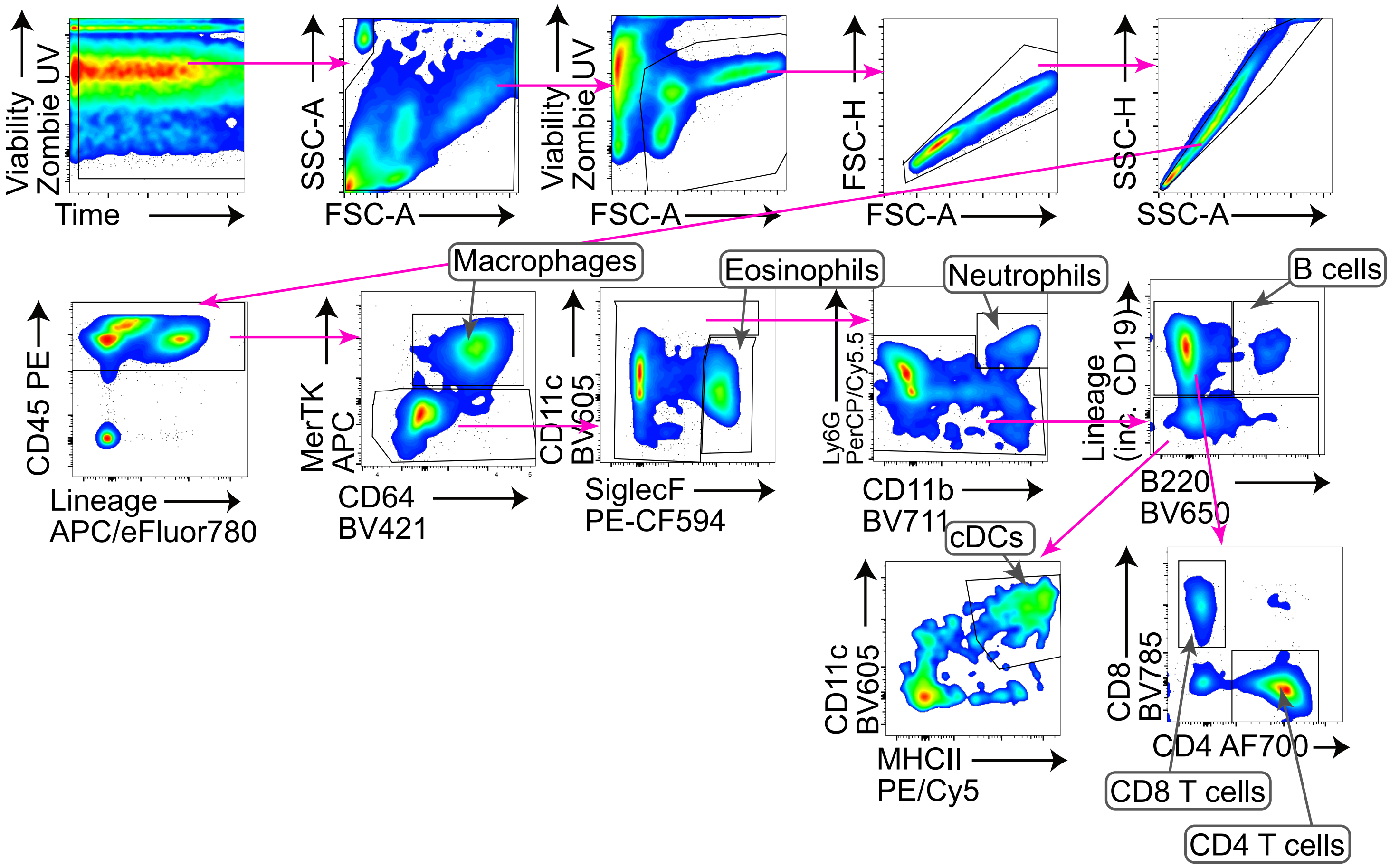

Supplementary Figure 13. Gating scheme murine BAL. BAL cell isolates were assessed via flow cytometry to define immune cell populations via the gating strategy shown. Lineage gate includes Ter119 APC-e780, CD3 APC-e780, TCRb APC-e780and CD19-APC-e780. Live, singlet, $CD45^+$ cells were assessed, prior to identifying $MerTK^+$ $CD64^+$ macrophages, $CD11b^+Ly6G^+$ neutrophils, $SiglecF^+$ eosinophils, $CD4^+$ and $CD8^+$ T cells.

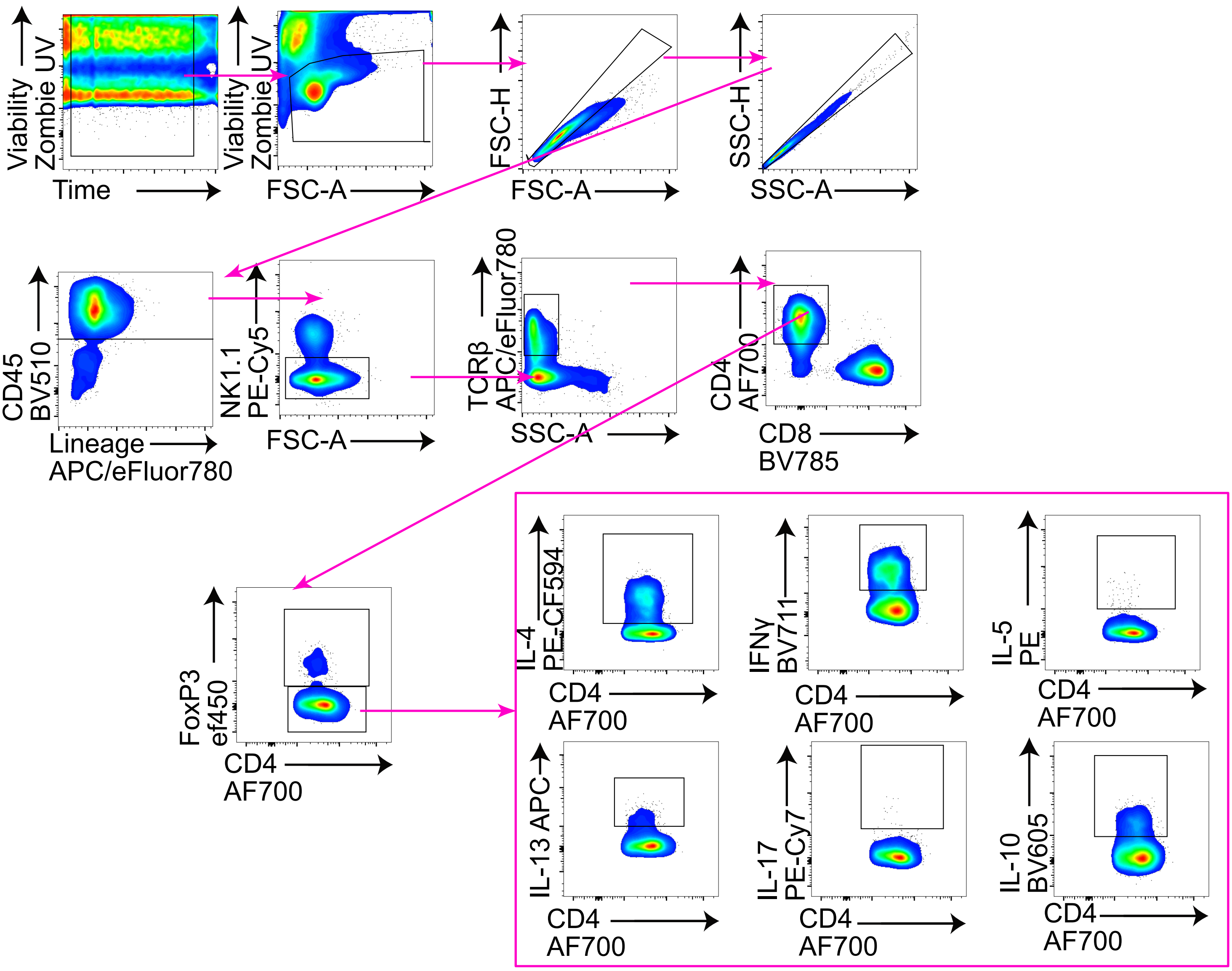

Supplementary Figure 14. Gating scheme murine lung T cell cytokines. Lung cell isolates were stimulated (PMA/ionomycin) and then assessed via flow cytometry to define immune cell populations via the gating strategy shown. Live, singlet, CD45^{+} cells were assessed, prior to identifying including CD4^{+} T cells expressing IL-4, IFNγ, IL-5, IL-13, IL-17 and IL-10.

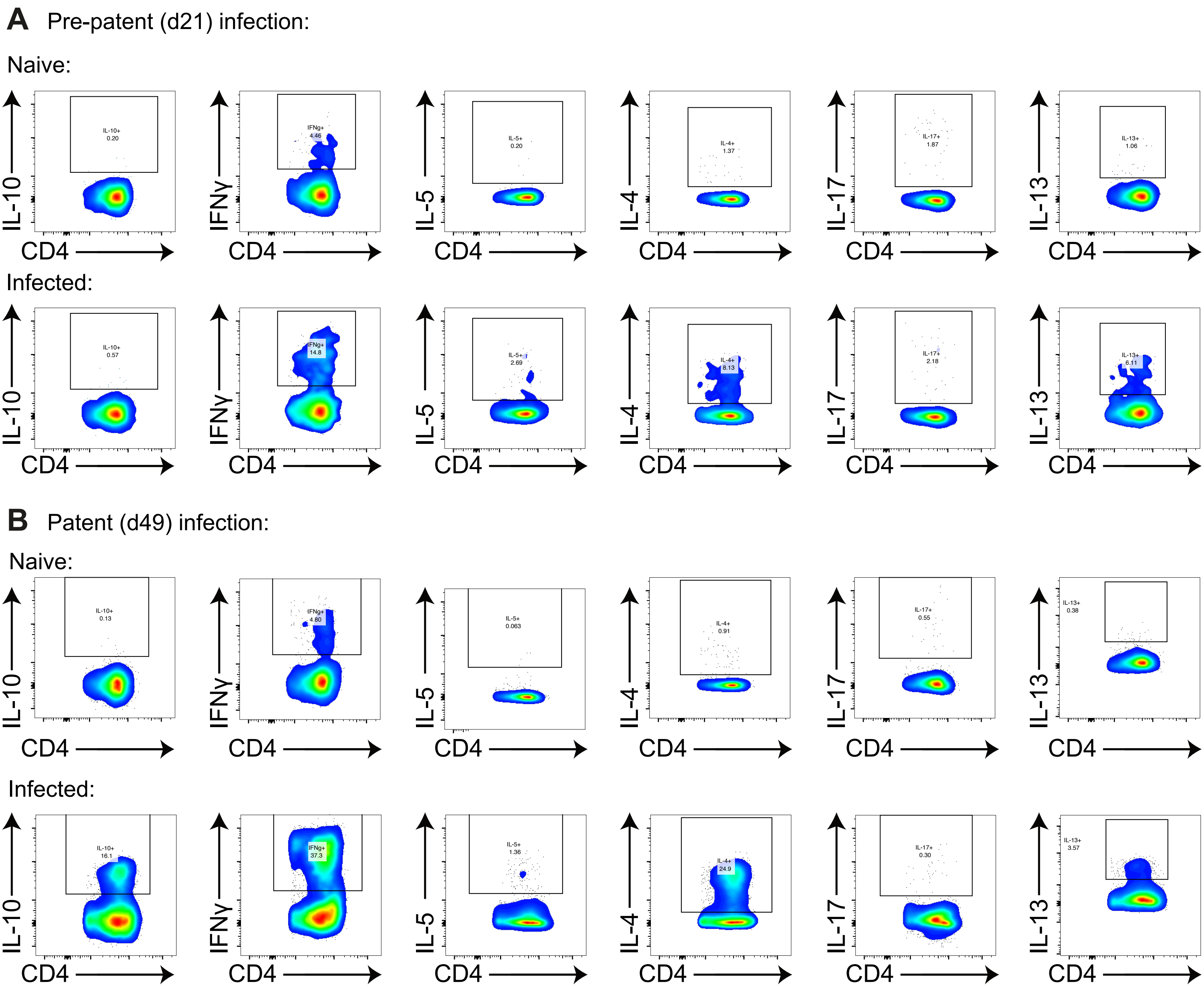

Supplementary Figure 15. Concatenated plots murine lung T cell cytokines. C57BL/6 mice were percutaneously infected with 180 cercariae, and lung samples taken at d21 (A) and d49 (B). Lung cell isolates were stimulated (PMA/ionomycin) and then assessed via flow cytometry to define immune cell populations via the gating strategy shown in Supplementary Figure 14. Plots are concatenated from one representative experiment at A) d21 or B) d49, with naive and infected mice separately concatenated. Fluorophores used designated in Supplementary Figure 14.

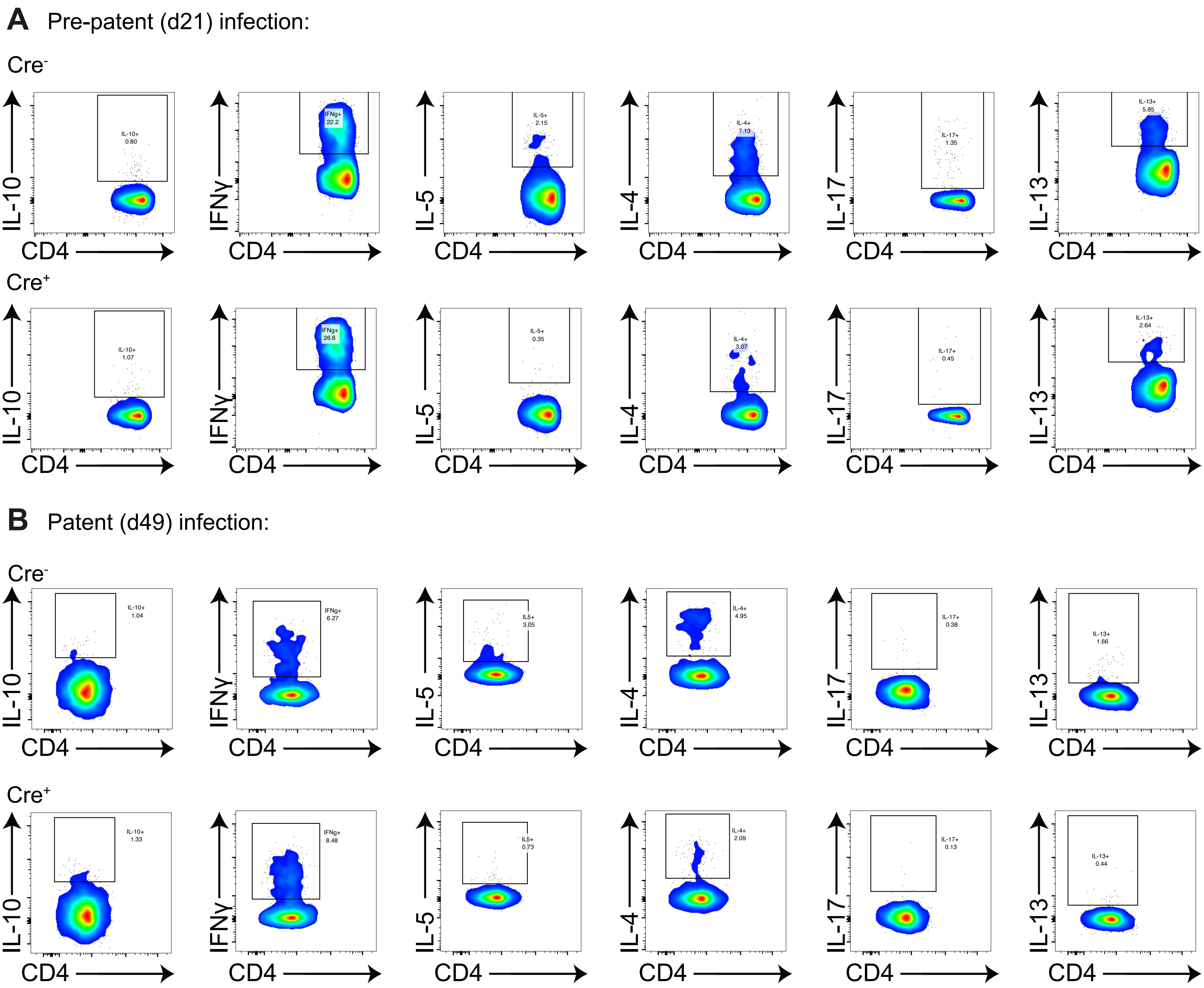

Supplementary Figure 16. Concatenated plots murine lung T cell cytokines. CD11cΔIrf4 mice were percutaneously infected with 180 cercariae, and samples taken at d21 (A) and d49 (B). Lung cell isolates were stimulated (PMA/ionomycin) and then assessed via flow cytometry to define immune cell populations via the gating strategy shown in Supplementary Figure 14. Plots are concatenated from one representative experiment at A) d21 or B) d49, with cre^+ and cre^- mice separately concatenated. Fluorophores used designated in Supplementary Figure 14.

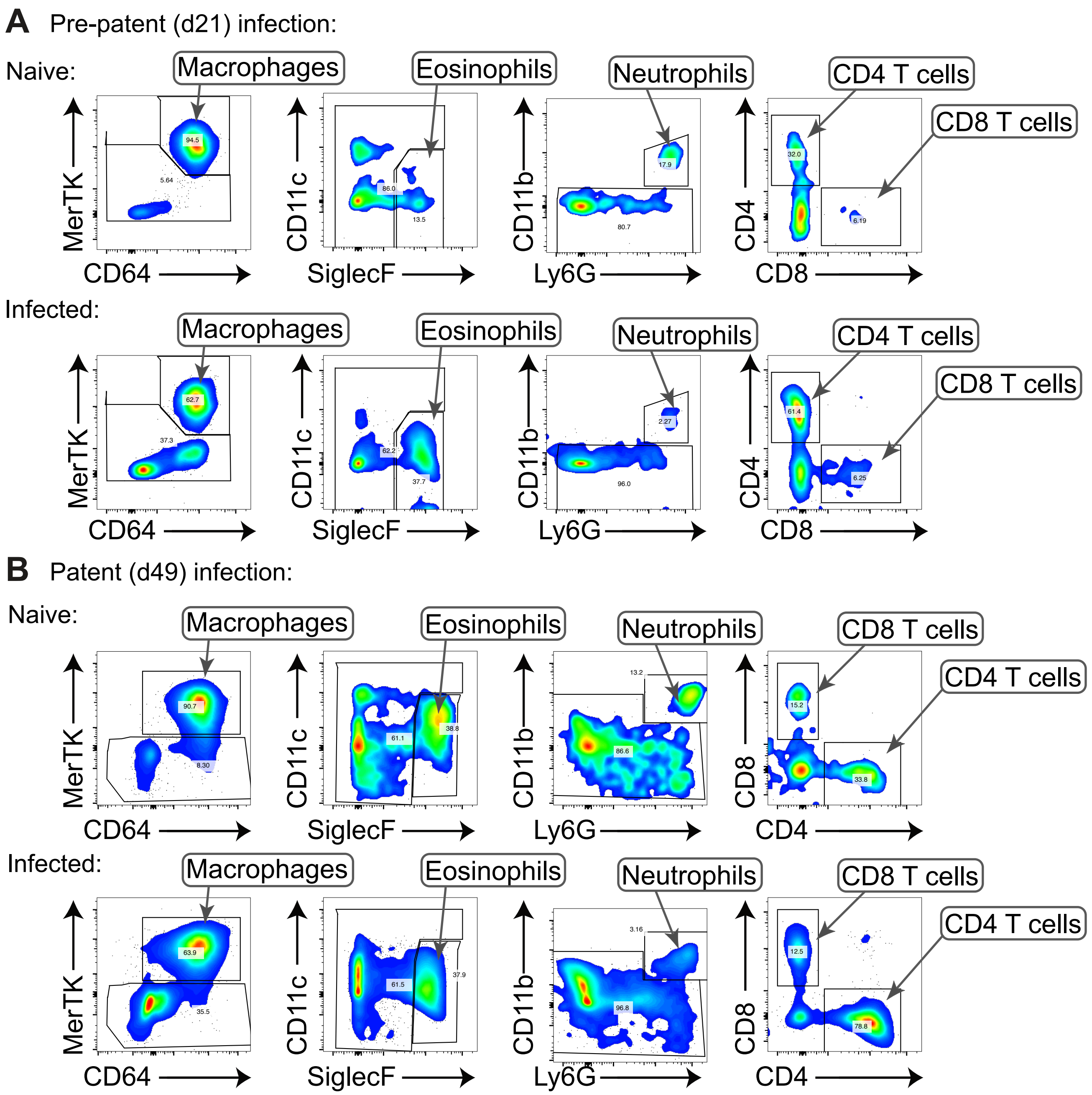

Supplementary Figure 17. Concatenated plots murine BAL. C57BL/6 mice were percutaneously infected with 180 cercariae, and BAL samples taken at d21 (A) and d49 (B). BAL cell isolates were assessed via flow cytometry to define immune cell populations via the gating strategy shown in Supplementary Figure 13. Plots are concatenated from one representative experiment at A) d21 or B) d49, with naive and infected mice separately concatenated. Fluorophores used designated in Supplementary Figure 13.

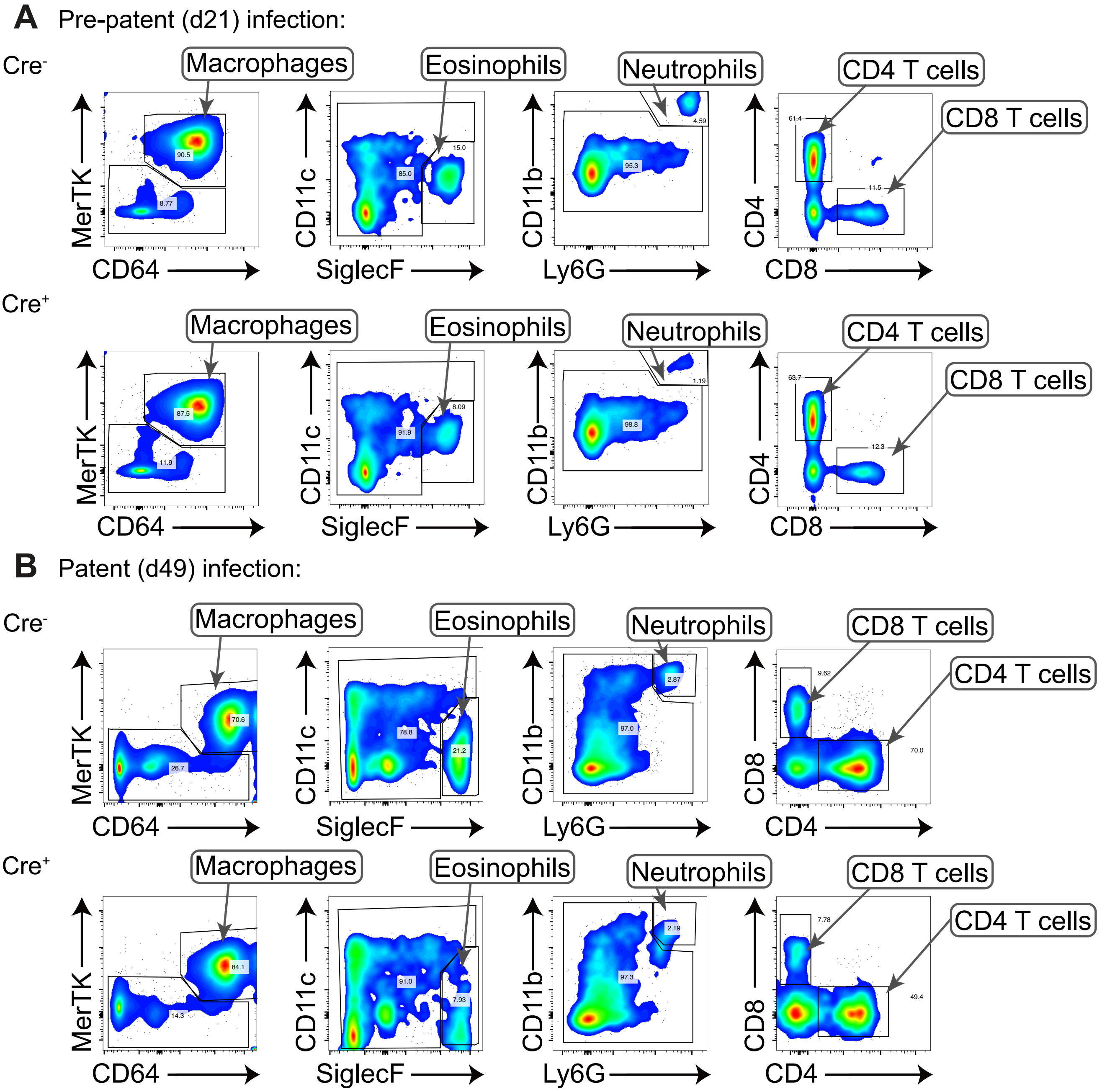

Supplementary Figure 18. Concatenated plots murine BAL. CD11cΔIrf4 mice were percutaneously infected with 180 cercariae, and samples taken at d21 (A) and d49 (B). BAL cell isolates were assessed via flow cytometry to define immune cell populations via the gating strategy shown in Supplementary Figure 13. Plots are concatenated from one representative experiment at A) d21 or B) d49, with cre^{+} and cre^{-} mice separately concatenated. Fluorophores used designated in Supplementary Figure 13.

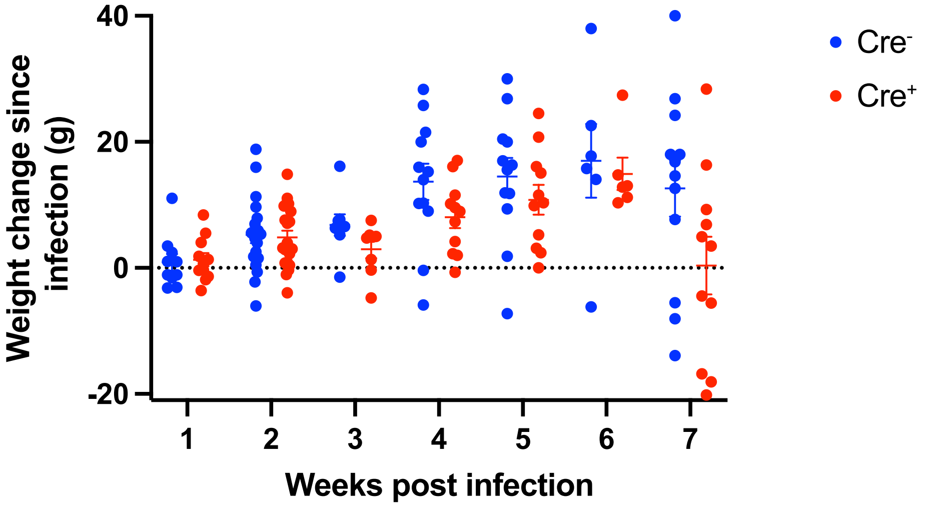

Supplementary Figure 19. Weight change post infection of CD11cΔIrf4 mice. CD11cΔIrf4 mice were percutaneously infected with 180 cercariae and weight monitored. Median weights per mouse within each week post infection are shown. Two Cre$^+$ mice were euthanised early (d43) due to reaching their severity limit (20% weight loss). Data are from 5 independent experiments (n=42 biologically independent animals in pre-patent infection stages, following this 19 mice were culled experimentally, leaving n=23 followed to week 7). Multiple two-sided T tests were used to compare Cre$^+$ and Cre$^-$ weights at each timepoint, no significant differences were observed. Data are presented as mean values +/- SEM. Source data are provided as a Source Data file.

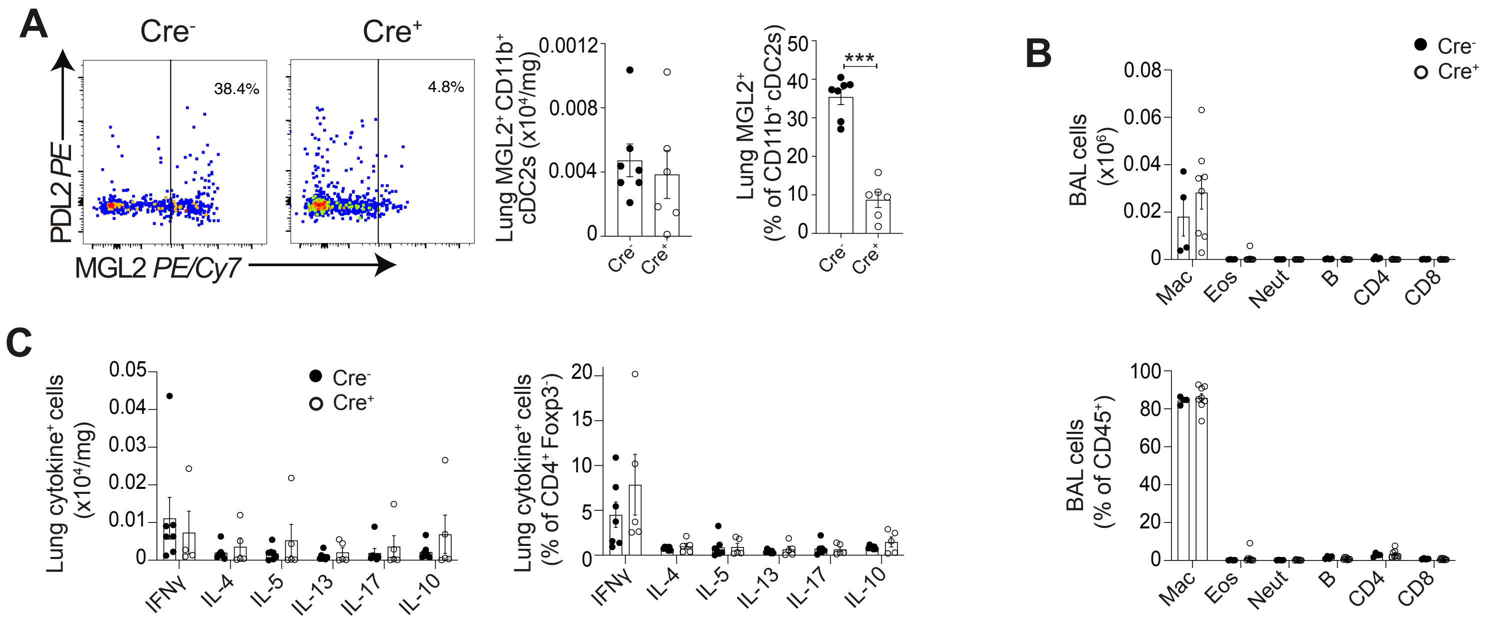

Supplementary Figure 20. Baseline (naive) characteristics of CD11cΔIrf4 mice. A) Representative flow cytometry plots show depletion of lung MGL2⁺ IRF4 dependent cDC2s. Gate frequencies show % of CD11b⁺ cDC2s. B) BAL cell isolates were assessed via flow cytometry for macrophages, eosinophils, neutrophils, B cells, CD4⁺ and CD8⁺ T cells. C) Lung cell isolates were stimulated with PMA/ionomycin, and cytokine production assessed via flow cytometry. Data are from 3 independent experiments, (n= 12 biologically independent animals). Data were fit to a linear mixed effect model, with experimental day as a random effect variable, and groups compared with a two-sided LS means students t test. Data are presented as mean values +/- SEM. Source data are provided as a Source Data file.

*=P <0.05, **=p<0.01, ***=p<0.001.

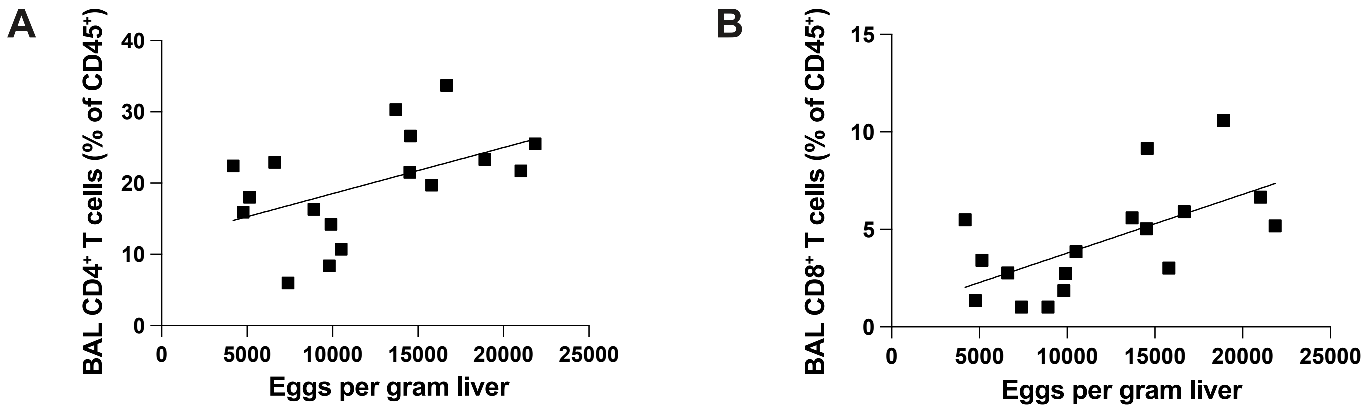

Supplementary Figure 21. Correlation BAL T cells and egg counts d49. Correlation between BAL T cell populations and egg counts was determined at d49 post infection. A non-parametric Spearman's correlation analysis was performed. A) $CD4^+$ T cells vs egg count, r = 0.4926, p=0.0465, n=17. B) $CD8^+$ T cells vs egg count, r = 0.6266, p=0.0084, n=17. Two tailed p values are presented. Source data are provided as a Source Data file.
