# Supplementary material for: Pulmonary inflammation promoted by type-2 dendritic cells is a feature of human and murine schistosomiasis
Source: Nat Commun. 2023 Apr 3;14:1863. doi: 10.1038/s41467-023-37502-z (PMC10070318; doi:10.1038/s41467-023-37502-z)
Supplement: Supplementary file 3 — Reporting Summary [file 41467_2023_37502_MOESM3_ESM.pdf]

nature portfolio

Corresponding author(s): Andrew S. MacDonald

Last updated by author(s): 27th February 2023

Reporting Summary

Nature Portfolio wishes to improve the reproducibility of the work that we publish. This form provides structure for consistency and transparency in reporting. For further information on Nature Portfolio policies, see our Editorial Policies and the Editorial Policy Checklist.

Statistics

For all statistical analyses, confirm that the following items are present in the figure legend, table legend, main text, or Methods section.

n/a	Confirmed	
☐	☒	The exact sample size (*n*) for each experimental group/condition, given as a discrete number and unit of measurement
☐	☒	A statement on whether measurements were taken from distinct samples or whether the same sample was measured repeatedly
☐	☒	The statistical test(s) used AND whether they are one- or two-sided<br>*Only common tests should be described solely by name; describe more complex techniques in the Methods section.*
☒	☐	A description of all covariates tested
☐	☒	A description of any assumptions or corrections, such as tests of normality and adjustment for multiple comparisons
☐	☒	A full description of the statistical parameters including central tendency (e.g. means) or other basic estimates (e.g. regression coefficient) AND variation (e.g. standard deviation) or associated estimates of uncertainty (e.g. confidence intervals)
☐	☒	For null hypothesis testing, the test statistic (e.g. *F*, *t*, *r*) with confidence intervals, effect sizes, degrees of freedom and *P* value noted<br>*Give P values as exact values whenever suitable.*
☒	☐	For Bayesian analysis, information on the choice of priors and Markov chain Monte Carlo settings
☒	☐	For hierarchical and complex designs, identification of the appropriate level for tests and full reporting of outcomes
☒	☐	Estimates of effect sizes (e.g. Cohen's *d*, Pearson's *r*), indicating how they were calculated

Our web collection on statistics for biologists contains articles on many of the points above.

Software and code

Policy information about availability of computer code

Data collection	BD FACSDiva and FlowJo (v10) for flow cytometry, Bio-Plex manager (v6.1) for Luminex analysis.
Data analysis	GraphPad Prism 7 and 9, RStudio (v2022.07.1+554) and R (v4.2.1)

For manuscripts utilizing custom algorithms or software that are central to the research but not yet described in published literature, software must be made available to editors and reviewers. We strongly encourage code deposition in a community repository (e.g. GitHub). See the Nature Portfolio guidelines for submitting code & software for further information.

Data

Policy information about availability of data

All manuscripts must include a data availability statement. This statement should provide the following information, where applicable:

- Accession codes, unique identifiers, or web links for publicly available datasets
- A description of any restrictions on data availability
- For clinical datasets or third party data, please ensure that the statement adheres to our policy

Source data underlying Figures and Supplementary Figures are provided with this paper in the Source Data file.

Human research participants

Policy information about studies involving human research participants and Sex and Gender in Research.

Reporting on sex and gender	Sex and gender was not considered in the study design due to the relatively low sample size of this work making this comparison underpowered.
Population characteristics	Pre-patent controlled human infection study:<br>Ages: 18-35 years<br>Sex: 2 Males, 1 Female<br>Endemic patent infection study:<br>Ages: 18-23 years<br>Sex: 15 males, 12 Females<br>Further characterisation in Supplementary Figure 2.
Recruitment	Participants were recruited via advertising (posters, instagram etc) for the controlled human infection study (Netherlands). Participants were recruited in person for the endemic patent infection study (Uganda), within a 2km radius of the Kigungu landing site controls from colleges, cases from the community, with the majority of the cases doing work involving regular lake contact.
Ethics oversight	The study protocols were approved by:<br>LUMC Institutional Medical Ethical Research Committee<br>UVRI Research Ethics Committee<br>Manchester University Research Ethics Committee<br>Ugandan National Council for Science and Technology

Note that full information on the approval of the study protocol must also be provided in the manuscript.

Field-specific reporting

Please select the one below that is the best fit for your research. If you are not sure, read the appropriate sections before making your selection.

☒ Life sciences ☐ Behavioural & social sciences ☐ Ecological, evolutionary & environmental sciences

For a reference copy of the document with all sections, see nature.com/documents/nr-reporting-summary-flat.pdf

Life sciences study design

All studies must disclose on these points even when the disclosure is negative.

Sample size	For all animal work between 4 and 16 mice were chosen per group. This sample size comes from at least 2 independent experiments. This sample size was chosen to be sufficient based upon initial experiments with this model, taking into account the variation in the system and the resultant power of the analyses to detect the primary outcomes of each experiment.<br>Multiple experiments with a small sample size were chosen to account for variation on the day of infection.<br>It was not possible to do prior power analysis for human studies, due to the novelty of this analysis we were unable to estimate required parameters. Our ethics allowed for the collection of sputum from up to 30 endemic patent infected individuals.
Data exclusions	For Cd11cIRF4 experiments, Cre+ mice were excluded when no evidence of depletion of Irf4 dependent MGL2+ cDC2s was observed. Similarly, Cre- littermates were excluded from analysis if <1% of their BAL cells were eosinophils at d21 post infection, as these were not representative of wild-type responses previously observed.
Replication	All murine experiments were performed in at least 2 independent repeats, with all experiments shown in the accompanying figure. Attempts at replication were successful.<br>Human experiments were not replicated in this study due to practical constraints on acquiring further samples and ethical limitations.
Randomization	Human samples were allocated to groups based upon schistosome infection status and were not randomised.<br>Murine samples: Mice were not formally randomized. For CD11cIRF4 experiments mice were genotyped in littermate cages and maintained in these cages throughout the experiment, with Cre+ and Cre- mice effectively randomly distributed within cages.
Blinding	Blinding was not used for this study. The study was not considered to be susceptible to the subjectivity of the researcher, with measurements and analysis techniques used applied equally to each group (flow cytometry gate positions for instance).

Reporting for specific materials, systems and methods

We require information from authors about some types of materials, experimental systems and methods used in many studies. Here, indicate whether each material, system or method listed is relevant to your study. If you are not sure if a list item applies to your research, read the appropriate section before selecting a response.

Materials & experimental systems

n/a	Involved in the study	
☐	☒	Antibodies
☒	☐	Eukaryotic cell lines
☒	☐	Palaeontology and archaeology
☐	☒	Animals and other organisms
☒	☐	Clinical data
☒	☐	Dual use research of concern

Methods

n/a	Involved in the study	
☒	☐	ChIP-seq
☐	☒	Flow cytometry
☒	☐	MRI-based neuroimaging

Antibodies

Antibodies used

Murine antibodies:
Antigen: B220, clone: RA3-6B2, fluorophore: BV650, titration: 1:100, manufacturer: BioLegend, catalogue number: 103241
Antigen: CD11b, clone: M1/70, fluorophore: BV711, titration: 1:1000, manufacturer: BioLegend, catalogue number: 101242
Antigen: CD11c, clone: N418, fluorophore: BV605, titration: 1:600, manufacturer: BioLegend, catalogue number: 117333
Antigen: CD19, clone: eBio1D3 (1D3), fluorophore: APC/e780, titration: 1:100, manufacturer: eBioscience, catalogue number: 47-0193-82
Antigen: CD16/32 (Fcblock), clone: 2.462, fluorophore: Purified, titration: 1:200, manufacturer: BD Biosciences, catalogue number: 553141
Antigen: CD3, clone: 17A2, fluorophore: APC/e780, titration: 1:100, manufacturer: eBioscience, catalogue number: 47-0032-82
Antigen: CD4, clone: RM4-5, fluorophore: AF700, titration: 1:200, manufacturer: BioLegend, catalogue number: 100536
Antigen: CD45, clone: A20, fluorophore: BV785, titration: 1:800, manufacturer: BioLegend, catalogue number: 110743
Antigen: CD45, clone: A20, fluorophore: PE, titration: 1:800, manufacturer: BioLegend, catalogue number: 110707
Antigen: CD64, clone: X54-5171, fluorophore: BV421, titration: 1:100, manufacturer: BioLegend, catalogue number: 139309
Antigen: CD8, clone: 53-6.7, fluorophore: BV785, titration: 1:200, manufacturer: BioLegend, catalogue number: 100750
Antigen: Foxp3, clone: FJK-16s, fluorophore: ef450, titration: 1:200, manufacturer: eBioscience, catalogue number: 48-5773-82
Antigen: IFN , clone: XMG1.2, fluorophore: BV711, titration: 1:200, manufacturer: BioLegend, catalogue number: 505835
Antigen: IL-10, clone: JESS-16E3, fluorophore: BV605, titration: 1:200, manufacturer: BioLegend, catalogue number: 505031
Antigen: IL-13, clone: W17010B, fluorophore: APC, titration: 1:200, manufacturer: eBioscience, catalogue number: 159405
Antigen: IL-17, clone: TC11-18H10, fluorophore: PE Cy7, titration: 1:200, manufacturer: BioLegend, catalogue number: 506922
Antigen: IL-4, clone: 11611, fluorophore: PE Dazzle" 594, titration: 1:200, manufacturer: BioLegend, catalogue number: 504132
Antigen: IL-5, clone: TRFK5, fluorophore: PE, titration: 1:200, manufacturer: BioLegend, catalogue number: 504304
Antigen: Ly6G, clone: 1A8, fluorophore: APC/Cy7, titration: 1:200, manufacturer: BioLegend, catalogue number: 127624
Antigen: Ly6G, clone: 1A8, fluorophore: PerCP Cy5.5, titration: 1:200, manufacturer: BioLegend, catalogue number: 127624
Antigen: MerTK, clone: 2BioI42, fluorophore: APC, titration: 1:100, manufacturer: BioLegend, catalogue number: 151508
Antigen: MGL2, clone: URA-1, fluorophore: PE Cy7, titration: 1:400, manufacturer: BioLegend, catalogue number: 146808
Antigen: MHCII, clone: M5/114.15.2, fluorophore: PE Cy5, titration: 1:20000, manufacturer: BioLegend, catalogue number: 107612
Antigen: NK1.1, clone: PK136, fluorophore: APC/e780, titration: 1:200, manufacturer: eBioscience, catalogue number: 47-5941-82
Antigen: NK1.1, clone: PK136, fluorophore: PE Cy5, titration: 1:200, manufacturer: BioLegend, catalogue number: 108716
Antigen: PDCA-1, clone: 927, fluorophore: BV650, titration: 1:200, manufacturer: BioLegend, catalogue number: 127019
Antigen: PDL2, clone: TY25, fluorophore: PE, titration: 1:200, manufacturer: BioLegend, catalogue number: 107206
Antigen: SiglecF, clone: E50-2440, fluorophore: PE CF594, titration: 1:400, manufacturer: BD Biosciences, catalogue number: 562757
Antigen: TCR , clone: H57-597, fluorophore: APC/e780, titration: 1:100, manufacturer: eBioscience, catalogue number: 47-5961-82
Antigen: Ter119, clone: TER-119, fluorophore: APC/e780, titration: 1:200, manufacturer: eBioscience, catalogue number: 47-5921-82
Antigen: XCR1, clone: 2ET, fluorophore: BV510, titration: 1:400, manufacturer: BioLegend, catalogue number: 107206
Human antibodies:
Antigen: CD3, clone: UCHT1, fluorophore: BB515, titration: 1:2000, manufacturer: BD Biosciences, catalogue number: 564465
Antigen: CD8, clone: RPA-T8, fluorophore: BB700, titration: 1:2000, manufacturer: BD Biosciences, catalogue number: 566452
Antigen: CD19, clone: H1B19, fluorophore: BB700, titration: 1:100, manufacturer: BD Biosciences, catalogue number: 745907
Antigen: CCR3, clone: 5E8, fluorophore: APC, titration: 1:200, manufacturer: BioLegend, catalogue number: 310708
Antigen: CD4, clone: RPA-T4, fluorophore: AF700, titration: 1:200, manufacturer: BioLegend, catalogue number: 300526
Antigen: CD11b, clone: ICRF44, fluorophore: AF700, titration: 1:200, manufacturer: BioLegend, catalogue number: 301355
Antigen: CD16, clone: 368, fluorophore: APC/Fire750, titration: 1:200, manufacturer: BioLegend, catalogue number: 980114
Antigen: CD28, clone: CD28.2, fluorophore: BV421, titration: 1:200, manufacturer: BioLegend, catalogue number: 102127
Antigen: CD1c, clone: L161, fluorophore: BV421, titration: 1:200, manufacturer: BioLegend, catalogue number: 331526
Antigen: CD45, clone: H130, fluorophore: BB515, titration: 1:200, manufacturer: BD Biosciences, catalogue number: 564585
Antigen: TCR , clone: IP26, fluorophore: BV605, titration: 1:200, manufacturer: BioLegend, catalogue number: 306732
Antigen: Siglec8, clone: 837535, fluorophore: BV605, titration: 1:200, manufacturer: BD Biosciences, catalogue number: 747872
Antigen: CD11c, clone: BU1S, fluorophore: BV650, titration: 1:50, manufacturer: BioLegend, catalogue number: 337238
Antigen: TCRgd, clone: 11F2, fluorophore: BV711, titration: 1:50, manufacturer: BD Biosciences, catalogue number: 568490
Antigen: CD14, clone: M5E2, fluorophore: BV711, titration: 1:200, manufacturer: BioLegend, catalogue number: 301837
Antigen: HLA-DR, clone: L243, fluorophore: BV785, titration: 1:200, manufacturer: BioLegend, catalogue number: 307642
Antigen: CD127, clone: A019DS, fluorophore: PE, titration: 1:200, manufacturer: BioLegend, catalogue number: 986002
Antigen: Clec9a, clone: 8F9, fluorophore: PE, titration: 1:100, manufacturer: BioLegend, catalogue number: 353803
Antigen: CD66b, clone: G1OFS, fluorophore: PE CF594, titration: 1:200, manufacturer: BioLegend, catalogue number: 305121
Antigen: CD7, clone: CD7-667, fluorophore: PE Cy5, titration: 1:100, manufacturer: BioLegend, catalogue number: 343110
Antigen: Foxp3, clone: 236A/E7, fluorophore: PE Cy7, titration: 1:200, manufacturer: eBioscience, catalogue number: 25-4777-42

Antigen: CD1a, clone: H1149, fluorophore: PE Cy7, titration: 1:200, manufacturer: BioLegend, catalogue number: 300122
Antigen: CD45, clone: H130, fluorophore: BV785, titration: 1:100, manufacturer: BioLegend, catalogue number: 304048
Antigen: HLA-DR, clone: L243, fluorophore: BV711, titration: 1:100, manufacturer: BioLegend, catalogue number: 307644
Antigen: CD16, clone: 3G8, fluorophore: BV510, titration: 1:100, manufacturer: BioLegend, catalogue number: 302048
Antigen: CD14, clone: M5E2, fluorophore: APC, titration: 1:50, manufacturer: BioLegend, catalogue number: 982506

Validation

Validation of antibodies was performed by manufacturers as below.

Biolegend antibodies:
Specificity testing of 1-3 target cell types with either single- or multi-color analysis (including positive and negative cell types).
Once specificity is confirmed, each new lot must perform with similar intensity to the in-date reference lot. Brightness (MFI) is evaluated from both positive and negative populations.
Each lot product is validated by QC testing with a series of titration dilutions.

BD antibodies:
The following BD antibodies were used: B220, CD16/32 (Fcblock), SiglecF, CD3, CD8, CD19, CD45, Siglec8, TCRgd"
Antibody specificity
The specificity is confirmed using multiple methodologies that may include a combination of flow cytometry, immunofluorescence, immunohistochemistry or western blot to test staining on a combination of primary cells, cell lines or transfectant models.
All flow cytometry reagents are titrated on the relevant positive or negative cells. To save time and cell samples for researchers, test size reagents are bottled at an optimal concentration with the best signal-to-noise ratio on relevant models during the product development. To ensure consistent performance from lot-to-lot, each reagent is bottled to match the previous lot MFI.

Thermofisher(eBiosceinces) antibodies:
Target specificity validation - helps ensure the antibody will bind to the correct target. Our antibodies are being tested using at least one of the following methods to ensure proper functionality in researcher's experiments.
Knockout—expression testing using CRISPR-Cas9 cell models
Knockdown—expression testing using RNAi to knockdown gene of interest
Independent antibody verification (IAV)—measurement of target expression is performed using two differentially raised antibodies recognizing the same protein target
Cell treatment—detecting downstream events following cell treatment
Relative expression—using naturally occurring variable expression to confirm specificity
Neutralization—functional blocking of protein activity by antibody binding
Peptide array—using arrays to test reactivity against known protein modifications
SNAP-ChIF"—using SNAP-ChIP to test reactivity against known protein modifications
Immunoprecipitation-Mass Spectrometry (IP-MS)—testing using immunoprecipitation followed by mass spectrometry to identify antibody targets

Animals and other research organisms

Policy information about studies involving animals; ARRIVE guidelines recommended for reporting animal research, and Sex and Gender in Research

Laboratory animals

This study used C57BL/6 mice, WT and Itgaxcrelrf4fl (referred to as Cd11cIRF4) (Caton, Smith-Raska and Reizis, 2007; Persson et al., 2013). For the maintenance of the lifecycle the Female Tuck Ordinary (HsdOla:TO; Envigo) mice strain was used. Mice were aged 6-21 weeks. Mice were housed in individually ventilated cages, with cage temperature at 23 degrees C , humidity at 54% and a 12hr light/ dark cycle.
Biomplara glabrata was used for the production of cercariae. These were kept in aquaria at 28-30 degrees C.

Wild animals

No wild animals were used in this study.

Reporting on sex

Findings relate to both sexes. Sex was not considered in study design Both sexes were included in all analyses.

Field-collected samples

No field collected samples were used in this study.

Ethics oversight

Murine work was performed under the terms of a UK Home Office approved Project License (P44492AC9) at the Biological Services Facility at the University of Manchester (UoM). All procedures were ethically reviewed, performed under license and in accordance with the UK Home Office ASPA 1986 and the GSK Policy on the Care, Welfare and Treatment of Animals as well as the European Union Animals Directive 2010/63/EU and were approved by UoM’s Animal Welfare and Ethical Review Body (AWERB).
For production of the parasite life cycle mice were maintained at Aberystwyth University (AU). All procedures performed on mice and invertebrates at Aberystwyth University adhered to the United Kingdom Home Office Animals (Scientific Procedures) Act of 1986 (project license P3BC46FD) as well as the European Union Animals Directive 2010/63/EU and were approved by AU’s AWERB.

Note that full information on the approval of the study protocol must also be provided in the manuscript.

Flow Cytometry

Plots

Confirm that:

☒ The axis labels state the marker and fluorochrome used (e.g. CD4-FITC).

☒ The axis scales are clearly visible. Include numbers along axes only for bottom left plot of group (a 'group' is an analysis of identical markers).

☒ All plots are contour plots with outliers or pseudocolor plots.

☒ A numerical value for number of cells or percentage (with statistics) is provided.

Methodology

Sample preparation	Cell isolation (murine). Single-cell suspensions were prepared using the following methods: BAL was collected by washing of the peritoneal cavity or lungs with PBS containing 2% FBS and 2 mM EDTA (Sigma). Lungs were processed via incubation at 37°C with 0.8 U/ml Liberase TL (Sigma) and 80 U/ml DNase I type IV (Sigma) in HBSS (Gibco), as previously described68. After 40 min the digestion was halted with PBS containing 2% FBS and 2 mM EDTA, and suspensions passed through 70 m cell strainers. Red blood cells (RBCs) were lysed using RBC lysis buffer (Sigma) and leukocytes then used for flow cytometry or stimulation. To assess cytokine secretion potential, lung cells were stimulated for three hours at 37C with 30 ng/ml PMA (Sigma), 0.5µg/ml ionomycin (Sigma) and 1µl/ml GolgiStop (BD) in X-vivo-15 (Lonza), supplemented with 1% L-glutamine (Gibco) and 0.1% -mercaptoethanol (Sigma). Stimulations were carried out in a 96 well u bottom plate, at 4 x105 cells per well in 200 µl final volume.<br>Flow cytometry (murine and human). For murine lung tissue, 1 x106 cells per sample were stained, while for murine BAL and human sputum the entire sample was stained. Samples were washed with PBS and stained for viability with ZombieUV or ZombieNIR (1:2,000; Biolegend). Samples were then blocked with 5 g/ml CD16/CD32 (2.462; BioLegend) or Human FcBlock (BD) in FACS buffer (PBS containing 2% FBS and 2 mM EDTA) before staining for surface markers at 4C for 30 min. After staining, cells were washed twice in FACS buffer and then fixed in 1% paraformaldehyde in PBS for 10 min at room temperature. For detection of intracellular proteins, cells were fixed with BD cytofix/cytoperm (BD) for 1 hour, then washed three times with lx eBioscience permeabilization buffer (Thermofisher) and antibodies to intracellular markers added for overnight staining.<br>Cell isolation (human). Sputum was induced via oral administration of 200 g Salbutamol, followed by inhalation of nebulised 4.5% NaCl for up to 4 minutes, after which sputum expectoration was attempted69. NaCl inhalation and attempted sputum expectoration was repeated until a sample was produced, up to 3 times within 30 minutes. Forced expiratory volume was monitored throughout the procedure, and sputum placed on ice prior to processing. Sputum plugs were isolated, weighed and a 4x volume of 0.1% dithiothreitol (Sigma) was added and shaken at room temperature for 15 min69. An equal volume of PBS was then added, and samples filtered through sequential 100 then 40 m cell strainers. Samples were centrifuged and supernatants and cells cryopreserved at -80C, with cells resuspended in freezing media (50% RPMI (Gibco) with 50% FCS (Sigma) and 10% DMSO (Sigma)) controlled freezing of sputum cells was obtained by use of a Mr Frosty freezing container (Nalgene).<br>Flow cytometry (murine and human). For murine lung tissue, 1 x106 cells per sample were stained, while for murine BAL and human sputum the entire sample was stained. Samples were washed with PBS and stained for viability with ZombieUV or ZombieNIR (1:2,000; Biolegend). Samples were then blocked with 5 g/ml CD16/CD32 (2.462; BioLegend) or Human FcBlock (BD) in FACS buffer (PBS containing 2% FBS and 2 mM EDTA) before staining for surface markers at 4C for 30 min. After staining, cells were washed twice in FACS buffer and then fixed in 1% paraformaldehyde in PBS for 10 min at room temperature. For detection of intracellular proteins, cells were fixed with BD cytofix/cytoperm (BD) for 1 hour, then washed three times with lx eBioscience permeabilization buffer (Thermofisher) and antibodies to intracellular markers added for overnight staining.
Instrument	BD Fortessa or LSRII flow cytometers
Software	Software FacsDiva (BD) software and FlowJo v10
Cell population abundance	N/A no sorting was performed
Gating strategy	For all samples initial an initial 'time' gate was used in which viability was placed against time and a period of stable acquisition was chosen. Live cells were then gated based upon Viability vs FSC-A. Singlets were then chosen based upon FSC-H vs FSC-A and SSC-H vs SSC-A. Next CD45 was used to distinguish leukocytes. Positive and negative gates were drawn based upon the data with gates drawn in low density regions "gaps" between the cell populations, for instance between CD4+ and CD4- cells. When possible Fluorescence Minus One (FMO) controls were used, or in the case of low cell number Fluorescence Minus Many (FMM) controls were used to set a lower limit for the gate.<br>Further gating strategies and FMO/FMM control examples are included in the supplementary information.

☒ Tick this box to confirm that a figure exemplifying the gating strategy is provided in the Supplementary Information.
